# Magnetic resonance imaging of the cirrhotic liver: diagnosis of hepatocellular carcinoma and evaluation of response to treatment – Part 2

**DOI:** 10.1590/0100-3984.2015.0140

**Published:** 2017

**Authors:** Miguel Ramalho, António P. Matos, Mamdoh AlObaidy, Fernanda Velloni, Ersan Altun, Richard C. Semelka

**Affiliations:** 1 Department of Radiology, University of North Carolina at Chapel Hill, Chapel Hill, NC, USA, and Hospital Garcia de Orta, Almada, Portugal; 2 Department of Radiology, University of North Carolina at Chapel Hill, Chapel Hill, NC, USA, and King Faisal Specialist Hospital and Research Center, Riyadh, Saudi Arabia; 3 Department of Radiology, University of North Carolina at Chapel Hill, Chapel Hill, NC, USA

**Keywords:** Magnetic resonance imaging, Liver cirrhosis, Image enhancement, Contrast media

## Abstract

In the second part of this review, we will describe the ancillary imaging features of hepatocellular carcinoma (HCC) that can be seen on standard magnetic resonance imaging (MRI) protocol, and on novel and emerging protocols such as diffusion weighted imaging and utilization of hepatocyte-specific/hepatobiliary contrast agent. We will also describe the morphologic sub-types of HCC, and give a simplified non-invasive diagnostic algorithm for HCC, followed by a brief description of the liver imaging reporting and data system (LI-RADS), and MRI assessment of tumor response following locoregional therapy.

## Resumo

Na segunda parte desta revisão descreveremos os achados de imagem auxiliares para o diagnóstico de carcinoma hepatocelular (CHC) e que podem ser observados num protocolo de ressonância magnética (RM) padrão e em protocolos emergentes que incluem imagens de difusão e aplicação de contrastes hepatoespecíficos/hepatobiliares. Descreveremos também os subtipos morfológicos de CHC e um algoritmo diagnóstico não invasivo simplificado para o CHC, seguido de uma breve descrição do *liver imaging reporting and data system* (LI-RADS) e avaliação por RM após terapêutica locorregional.

## ANCILLARY IMAGING FEATURES FOR THE DIAGNOSIS OF HEPATOCELLULAR CARCINOMA DEPICTED IN STANDARD PROTOCOL

The American Association for the Study of Liver Diseases (AASLD) and European Association for the Study of the Liver (EASL) have validated imaging criteria for the diagnosis of hepatocellular carcinoma (HCC) in cirrhotic patients, which is based on arterial-phase hyper-enhancement relative to the background liver parenchyma and venous/equilibrium phase washout. However, as was stated in part 1 of this review, not all HCCs exhibit these characteristics.

In addition to arterial hyper-enhancement and delayed washout, which are the main features for the diagnosis of HCC, some ancillary signs have been described. Although none of them is specific of HCC in isolation, their presence increases the probability of HCC^(1)^. Interestingly, most of these features are depicted with magnetic resonance imaging (MRI).

**Figure 1:**
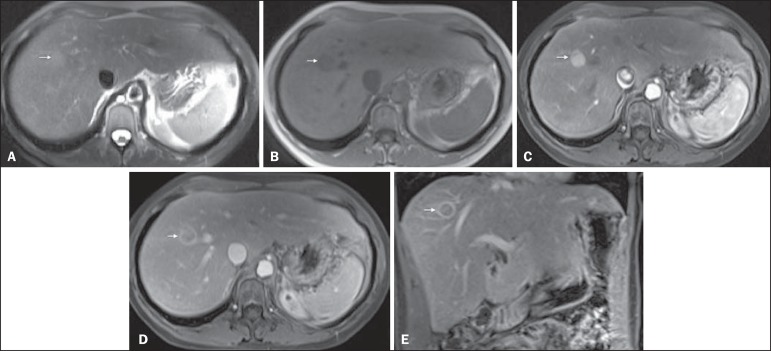
*Typical HCC in a patient with chronic hepatitis-C*. Axial fat-suppressed SS-FSE T2-WI (**A**), axial in-phase precontrast (**B**) and postcontrast fat-suppressed 3D-GRE T1-WI in the arterial (**C**) and interstitial (**D,E**) phases. A nodule with 2 cm is depicted on the right hepatic lobe (arrows, **A–E**), showing mild high signal intensity on T2-WI (**A**) and low-signal intensity on pre-contrast T1-WI (**B**). On the dynamic postcontrast images, the lesion shows arterial hyper-enhancement (**C**) and delayed washout with pseudocapsule enhancement (**D,E**). These features are diagnostic of HCC.

Delayed capsular enhancement is defined as a persistent peripheral hyper-enhancing rim seen in the delayed phase of enhancement (Figure 1), and could be helpful in lesions that do not show classical features of HCC on dynamic imaging^(2)^. Capsular enhancement has high specificity for HCC reportedly ranging from 83–96%^(3,4)^; however, sensitivity is only moderate, ranging from 43–55%^(3,4)^.

**Figure 2:**
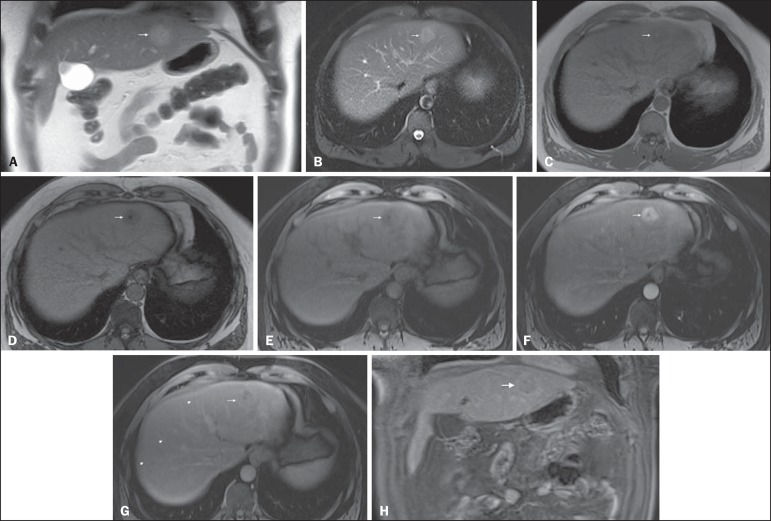
*Fatty HCC in a patient with non-alcoholic fatty liver disease*. Coronal SS-FSE T2-WI (**A**), axial fat-suppressed FSE T2-WI (**B**), axial in- (**C**) and out-of-phase (**D**) GRE T1-WI, axial pre- (**E**) and postcontrast fat-suppressed 3D-GRE T1-WI in the arterial (**F**) and interstitial (**G**) phases, and coronal fat-suppressed 3D-GRE T1-WI in the interstitial phase (**H**). One nodule is depicted on the left hepatic lobe (arrows, **A–G**), showing mild high signal intensity on T2-WI (**A,B**), low-signal intensity on in-phase T1-WI (**C**), and heterogeneous drop of signal on out-of-phase T1-WI (**D**). On the dynamic postcontrast images, the nodule is hypervascular (**F**) and shows delayed washout and pseudocapsule enhancement (**G,H**). Note the fine fibrotic bands of the liver parenchyma, which are seen at the late interstitial phase (arrowheads, **G**).

Intratumoral lipid is a relatively uncommon characteristic observed with HCC histologically (sensitivity for HCC of 12–37%)^(3,5-8)^, and is more commonly present in the form of intracellular lipid. Fatty metamorphosis may be identified in a subset of cases with chemical shift imaging (Figure 2), in form of loss of signal intensity on the out-of-phase images compared with the in-phase images. Conversely, lipid content is moderately specific for HCC (68–100%)^(3,5-8)^ and as stated above, any lipid-containing tumor in a cirrhotic liver should be viewed with suspicion, especially when the lesion is > 15 mm^(8)^.

The appearance of HCC on T2-weighted images is variable. Mild to moderate T2 hyperintensity is highly suggestive of malignancy if present. Differential diagnosis includes: early HCC, progressed HCC, and intrahepatic cholangiocarcinoma. The main limitation is the somewhat limited sensitivity for HCC, as many HCCs are T2 isointense or hypointense^(9)^. HCCs tend to show minimal to mildly increased signal intensity on T2-weighted images with a specificity and a positive predictive value for HCC varying from 73–100% and 72–100%, respectively^(3,5-7,10)^.

**Figure 3:**
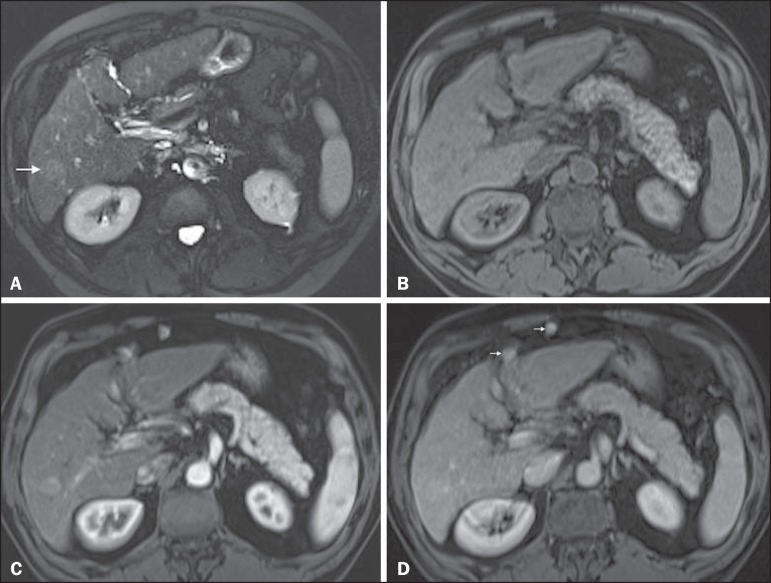
*HCC in a patient with chronic alcoholic liver disease*. Axial fat-suppressed SS-FSE T2-WI (**A**), axial pre- (**B**) and postcontrast fat-suppressed 3D-GRE T1-WI in the arterial (**C**) and interstitial (**D**) phases. There is a 2-cm lesion in the right hepatic lobe, showing mild high signal intensity on T2-WI (arrow, **A**), low signal on T1-WI, arterial hyper-enhancement with no washout on the delayed phase. Despite this lesion cannot be categorized as HCC by imaging criteria, the combination of mild high T2 signal intensity and hypervascular characteristics are very likely related to HCC in the setting of liver cirrhosis. Note the recanalization of the umbilical vein (arrows, **D**).

The elevated T2 signal in a focal lesion can be useful to reliably differentiate HCC from ndysplastic nodules (Figure 3)^(11)^. Recent studies have shown that the addition of T2-weighted imaging to gadolinium-enhanced T1-weighted 3D-GRE dynamic imaging improves the diagnostic performance of MRI in the detection of HCC compared to isolated dynamic MRI. This is especially helpful for lesions smaller than 10 or 20 mm, which may show hypervascularity, but might not show any washout^(12)^, increasing the suspicion for HCC^(13)^ (Figure 3).

## ANCILLARY IMAGING FEATURES FOR THE DIAGNOSIS OF HCC DEPICTED USING NOVEL AND EMERGING PRACTICES

### Diffusion-weighted imaging (DWI)

DWI is an imaging technique based on differences in the Brownian motion (diffusion) of water molecules within tissues. In highly cellular tissues such as in tumors (and resultant compression of the extracellular spaces), the water molecules diffusion is restricted. Hence, signal hyperintensity within HCC relative to liver parenchyma is expected^(14)^. The diffusion restriction can be confirmed by generating parametric apparent diffusion coefficient (ADC) maps showing lower ADC values than the adjacent liver.

A limited number of studies have shown encouraging results suggesting that DWI has a good diagnostic performance in the detection of HCC in patients with chronic liver disease and equivalent to conventional contrast-enhanced for lesions greater than 20 mm in size^(15,16)^. Currently, the limitation of DWI is primary lesion characterization rather than lesion detection^(15,16)^.

**Figure 4:**
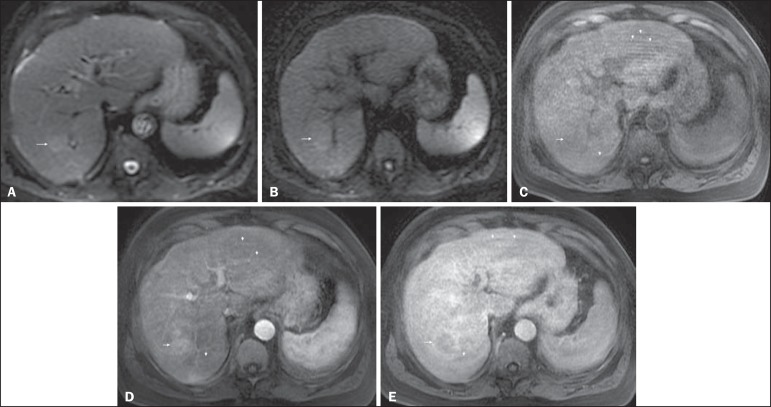
*HCC in a patient with chronic hepatitis-C unable to fully cooperate with the recommended breath-holds on the dynamic GRE sequences.* Axial DWI b = 50 s/m^2^ (**A**) and DWI b = 600 s/m^2^ (**B**), axial pre- (**C**) and postcontrast fat-suppressed 3D-GRE T1-WI in the arterial (**D**) and interstitial (**E**) phases. One nodule on the right hepatic lobe is depicted (arrow, **A–E**), showing mild high-signal intensity on DWI (**A,B**). On the dynamic postcontrast images, the lesion shows arterial hyper-enhancement (**D**), and shows delayed washout and pseudocapsule enhancement (**E**). Note that the diagnosis of HCC is confident, despite the low quality images due to respiratory motion artifacts (arrowheads, **C–E**).

The greatest benefit relies on the combined use of DWI with conventional dynamic MRI^(17,18)^ (Figure 4). A recent meta-analysis by Wu et al.^(16)^ found that DWI combined with conventional dynamic contrast-enhanced MRI performed significantly better than either DWI alone or conventional dynamic contrast-enhanced MRI alone (pooled sensitivity and specificity: 93% and 84% combined, 81% and 89% DWI, 79% and 62% dynamic contrast-enhanced ). Consequently, an additional acquisition of DWI is being implemented in abdominal protocols.

In a recent study, a new MRI criteria was proposed, combining the features of lesions after gadolinium-based contrast media administration and hyperintensity on DWI^(19)^. This significantly improved the sensitivity for the diagnosis of HCC compared to conventional hemodynamic criteria alone, irrespective of tumor size. However, additional larger studies are required to determine its role for the detection of HCC in patients with chronic liver diseases.

### Hepatocyte-specific/hepatobiliary contrast agents

MRI hepatobiliary contrast agents (HCAs) are shifting the paradigm of the diagnosis of HCC. Several recent studies have investigated the use of the hepatobiliary phase of hepatocyte-specific contrast agents for diagnosing HCC with promising results^(20,21)^. Two HCAs are currently available: gadoxetate disodium (Eovist^®^/Primovist^®^; Bayer Healthcare) and gadobenate dimeglumine (MultiHance^®^; Bracco Diagnostics)^(22)^. These two HCAs combine extracellular properties with liver-specific properties, allowing both dynamic and hepatobiliary imaging^(23)^. Gadoxetic acid is more highly liver-specific with approximately 50% of the injected dose taken up by functioning hepatocytes and is excreted in bile, allowing delayed uptake imaging within 20 min from the time of injection, compared with an uptake of 3–5% for gadobenate dimeglumine, which allows for delayed uptake imaging within 2–3 hours^(22)^. It is worth noting that the injection dose of gadoxetate disodium is smaller than that of extracellular gadolinium agents. This small dose can result in acquisition timing error and truncation artifacts in the arterial phase if not properly timed.

**Figure 5:**
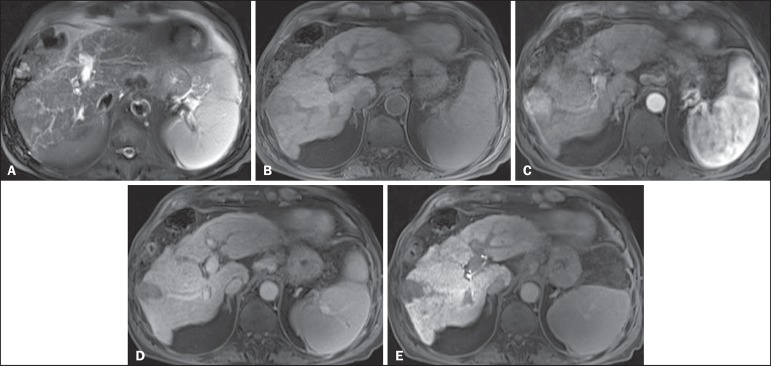
*HCC evaluated using gadoxetate disodium (hepatobiliary contrast agent).* Axial fat-suppressed (**A**) SS-FSE T2-WI, axial pre- (**B**) and postgadolinium (gadoxetate disodium) fat-suppressed 3D-GRE T1-WI in the arterial (**C**), interstitial (**D**) and hepatobiliary phases (**E**). An HCC is depicted in the right liver lobe, in a background cirrhotic parenchyma, showing mild high signal intensity on T2-WI, low signal intensity on T1-WI, hypervascular characteristics (**C**) and washout on the delayed phase (**D**). On the hepatobiliary phase, due to the presence of impaired hepatocytes, the HCC shows no enhancement (**E**). Note the enhancement of the biliary duct (arrowheads, **E**).

Using fluoroscopic triggering, with a low injection rate of 1 mL/s, to stretch the bolus or diluting the contrast with normal saline to 20 mL to enable a rapid injection rate at 2 mL/s are suggested solutions^(24-26)^. Hepatobiliary phase images are easy to recognize because both the liver and the bile ducts are markedly enhanced. The blood vessels as well as all non-hepatocellular lesions and lesions with impaired hepatocytes all appear hypointense. Typically, HCCs exhibit hypointensity on hepatobiliary phase images (Figure 5), except for some well-differentiated HCCs that may retain contrast.

The combination of routine dynamic and hepatobiliary imaging has been reported to be both sensitive and specific for HCC (sensitivity, 67–97%; specificity, 83–98%)^(5,7,27-33)^. Two recent meta-analyses found a pooled sensitivity of 91% and specificity of 93%^(21,34)^. The addition of hepatobiliary phase images improves the per-lesion sensitivity for the diagnosis of HCC by 6–15% for gadoxetate acid^(28,35,36)^ and by 9% for gadobenate dimeglumine^(37)^.

**Figure 6:**
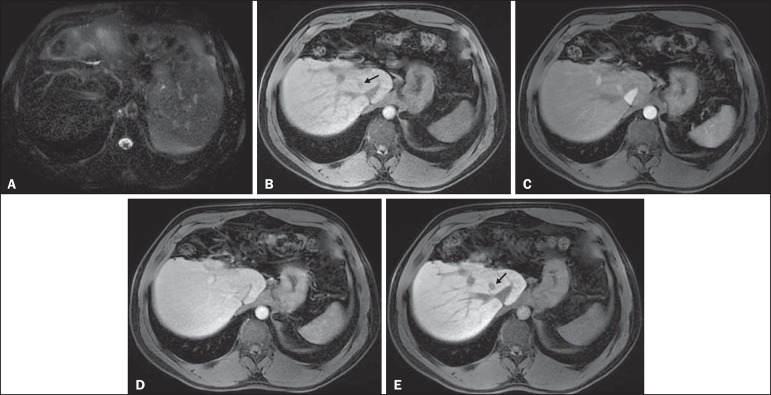
*Small HCC diagnosed using gadoxetate disodium (hepatobiliary contrast agent).* Axial fat-suppressed (**A**) SS-FSE T2-WI, axial pre- (**B**) and postgadolinium (gadoxetate disodium) fat-suppressed 3D-GRE T1-WI in the arterial (**C**), interstitial (**D**) and hepatobiliary phases (**E**). A small HCC is depicted in the right liver lobe, medial to the right hepatic vein, showing isointensity on T2-WI, mild low signal intensity on T1-WI, arterial hyper-enhancement (**C**) and no perceptible washout on the delayed phase (**D**). On the hepatobiliary phase, due to the presence of impaired hepatocytes, the HCC shows no enhancement (arrow, **E**). This case exemplifies the advantages of hepatobiliary contrast agents in the characterization of liver nodules in the setting of cirrhosis.

Gadoxetic acid-enhanced MRI has numerous advantages in imaging the cirrhotic liver including: i) higher sensitivity for the diagnosis of HCC, especially for lesions ≤ 20 mm (Figure 6)^(28,35,38,39)^; ii) improved characterization of arterially enhancing lesions without definite washout on subsequent imaging (Figure 6)^(33,35)^; iii) distinguishing arterially enhancing pseudo-lesions from HCC^(33)^; and iv) detection of lesions that are isointense to the background hepatic parenchyma on all sequences, apart from the hepatobiliary phase, that are at high risk of transforming to hypervascular HCC^(40,41)^. Nodules that show hypointensity on the hepatobiliary phase, but lacking diagnostic features of HCC on the earlier post-contrast phases may represent high-grade dysplastic nodules or early HCC^(6,7,42)^, and are at increased risk of progression to invasive hypervascular HCC.

### Morphologic HCC sub-types

**Figure 7:**
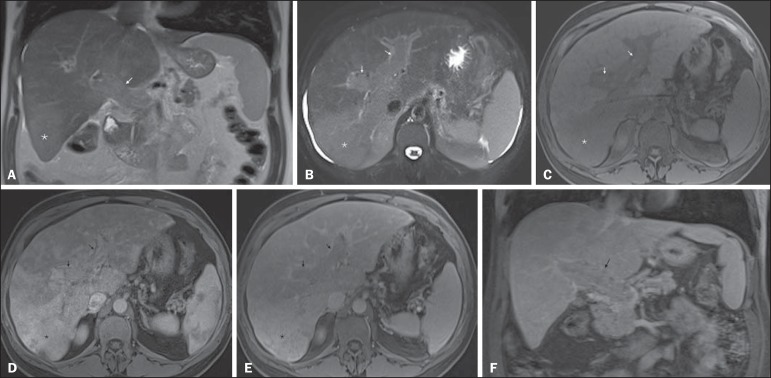
*Diffuse HCC in a patient with chronic hepatitis C.* Coronal SS-FSE T2-WI (**A**), axial fat-suppressed SS-FSE T2-WI (**B**), axial pre- (**C**) and postcontrast fat-suppressed 3D-GRE T1-WI in the arterial (**D**) and interstitial (**E**) phases, and coronal postcontrast fat-suppressed 3D-GRE T1-WI in the interstitial phase (**F**). A diffuse area of mild high-signal intensity on T2-WI (asterisk, **A,B**) is depicted on the right liver lobe, showing low-signal intensity on pre-contrast T1-WI (**C**). On the dynamic postcontrast images, the lesion is hypervascular at the arterial phase (**D**) and shows delayed heterogeneous mottled washout (**E,F**). These features are diagnostic of diffuse HCC. Note the tumor thrombus filling and expanding the portal vein, typical of this type of HCC (arrows, **A–F**). The thrombus shows hypervascular characteristics and delayed washout comparable to the MRI dynamic features of the tumor.

**Figure 8:**
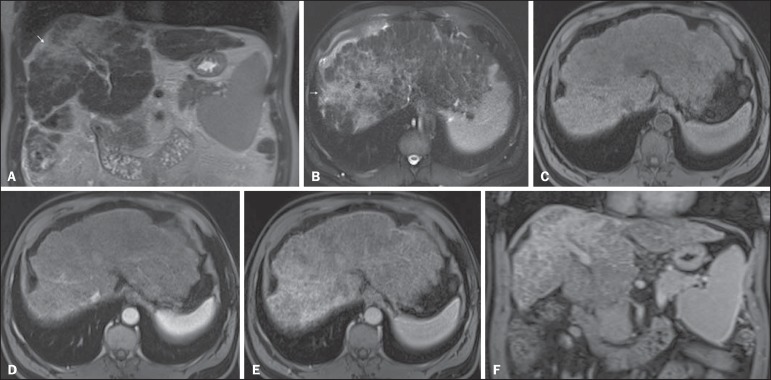
*Liver cirrhosis with confluent fibrosis.* Coronal (**A**) and axial fat-suppressed (**B**) SS-FSE T2-WI, axial pre- (**C**) and postcontrast fat-suppressed 3D-GRE T1-WI in the arterial (**D**) and interstitial (**E**) phases, and coronal postcontrast fat-suppressed 3D-GRE T1-WI in the interstitial phase (**F**). There is a linear pattern of fibrosis throughout the liver, with a focal region of confluent fibrosis in segments 7 and 8 peripherally (arrow, **A,B**), that is moderately high in signal on T2-WI (**A,B**) and mildly low in signal on T1-weighted image (**C**) and demonstrates negligible enhancement on early postcontrast image (**D**) and moderate enhancement on delayed image (**E,F**). The fine pattern of fibrosis is better depicted on late postgadolinium images as linear enhancing structures (**E,F**). Note the distorted anatomy and capsular retraction of segment 7, in relation to the more prominent region of fibrosis. The absence of portal vein thrombus, lack of arterial hyper-enhancement and delayed progressive enhancement allows the confident diagnosis of confluent fibrosis and excludes diffuse HCC.

HCCs can manifest as: i) focal (nodular); ii) massive; and iii) diffuse/infiltrative^(43)^. Nodular type is the most common encountered type and can be further classified as solitary or multi-focal. Multi-focal nodular subtype is an advanced and aggressive subtype and shows similar features to solitary nodular subtype on conventional and dynamic MRI. Additional features that are relatively uncommon with solitary focal lesions, but are noticed with multi-focal HCC and other aggressive subtypes include portal venous thrombosis and in intrahepatic metastases^(43)^. Massive tumors are large tumors that often render these patients non-eligible for locoregional ablative therapies or hepatic transplantation. Diffuse HCCs are usually large and have ill-defined boundaries without clear demarcation. They usually present with extremely high alpha-fetoprotein levels and are invariably associated with portal venous thrombus, which is almost always tumoral in nature. Diffuse HCCs can be extremely subtle, and therefore challenging to reach an early diagnosis as they can blend with the background liver parenchyma. Kneuertz et al.^(43)^ evaluated 147 patients with advanced HCCs (75 with infiltrative HCC and 72 patients with multi-focal HCC). In that study, failure to exhibit a distinct mass was observed in 42.7% of patients, whereas low T1 signal intensity was observed in 55.7% and high T2 signal intensity was observed in 80.3% of patients. They also showed mild miliary pattern of enhancement on the arterial phase imaging in 16.4% of patients, with delayed washout in 50.8% (Figure 7). In clinical practice, it has to be differentiated from areas of confluent fibrosis. Post-contrast delayed imaging is crucial as it demonstrates heterogeneous washout in diffuse HCC^(44)^, whereas confluent fibrosis shows increase enhancement over time (Figure 8). Another distinctive feature from confluent fibrosis is the presence of regional tumor thrombus that is almost invariably present in patients with diffuse HCC and uncommon in confluent fibrosis^(45)^.

A rare variant of nodular morphologic subtype is arterially rim-enhanced HCC. These tumors tend to present a more aggressive behavior with rapid interval growth and disease worsening^(46)^.

### Simplified schematic representation of focal lesions in the cirrhotic liver

**Figure 9:**
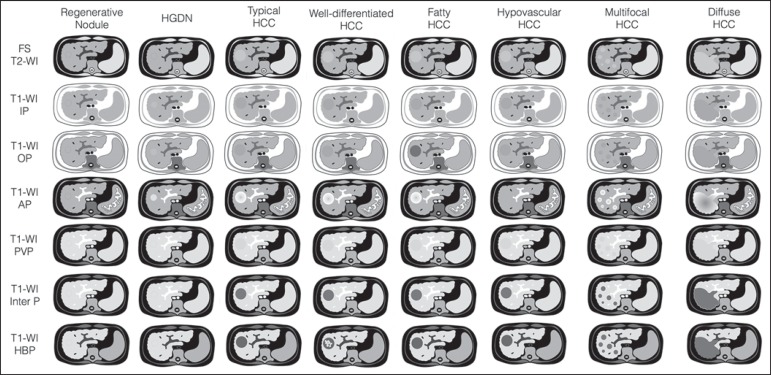
*Stereotypical simplified schematic representation, showing MRI features of cirrhotic nodules.* In this schematic representation it is shown the appearance of common hepatocellular nodules in the cirrhotic liver, using a standard abdominal protocol. Abbreviations: HCC, hepatocellular carcinoma; HGDN, high grade dysplastic nodule; FS T2-WI, fat-suppressed T2-weighted image; T1-WI IP, T1-weighted in-phase image; T1-WI OP, T1-weighted out-of-phase image; T1-WI AP, post-contrast fat-suppressed T1-weighted image at the late arterial phase; T1-WI PVP, post-contrast fat-suppressed T1-weighted image at the portal-venous phase; T1-WI Inter P, post-contrast fat-suppressed T1-weighted image at the interstitial phase; T1-WI HBP, post-contrast fat-suppressed T1-weighted image at the hepatobiliary phase (with hepatobiliary contrast agent).

A simplified schematic representation of the typical imaging features of the most common cirrhotic lesions is provided in Figure 9.

### Liver imaging reporting and data system (LI-RADS)

LI-RADS is an initiative supported and developed by the American College of Radiology (ACR)^(47)^ to improve terminology standardization and consensus for interpreting and reporting imaging findings of the liver in patients with cirrhosis or in at-risk for HCC. It has been developed to provide a framework for assigning degrees of concern on imaging findings, improving communication with clinicians, and facilitating decision making and outcome monitoring^(48)^. This evolving comprehensive document can be accessed online at the ACR website^(47)^. A detailed summary of LI-RADS is beyond the scope of this article.

The LI-RADS classifies lesions into five categories ranging from definitely benign to definitely HCC: LR-1 (definitely benign); LR-2 (probably benign); LR-3 (indeterminate probability of HCC); LR-4 (probably HCC); LR-5 (definitely HCC); and LR-M (other malignancy such as cholangiocarcinoma).

Western and Asian societies guidelines address the management of lesions that are definitely HCC by imaging criteria, i.e., wash-in/washout not taking in account ancillary imaging findings that are already used in routine clinical practice. LI-RADS expands the "indeterminate" category into probably benign, intermediate probability of HCC, and probably HCC (LI-RADS categories 2, 3 and 4). A LI-RADS category of 2, 3, or 4 should be issued along with a diagnostic recommendation, to assist reach greater diagnostic certainty^(49)^. This recommendation may include to: continue routine surveillance, repeat imaging at shorter than routine interval (short-time follow-up), or repeat imaging using a different method (alternative imaging), and/or engage in multi-disciplinary discussion^(48)^.

LI-RADS consider major features of HCC: A mass measuring ≥ 10 mm is characterized as HCC (LR-5) if it shows arterial hyper-enhancement and one of the following: i) washout feature and/or capsule enhancement (both features for lesions measuring 10 to < 20 mm and only one for lesions measuring ≥ 20 mm); ii) threshold growth (LR-5 g): diameter increase ≥ 50% in 6 months or ≥ 100% in > 6 months (absolute minimum growth of 5 mm required); iii) visualization on screening US of a ≥ 10 mm nodule that shows arterial hyper-enhancement and washout feature (LR-5us)^(49)^.

In the absence of typical enhancement features, ancillary features suspicious for malignancy, such as high T2 signal intensity, restricted diffusion, intra-lesional fat, hypointensity on hepatobiliary phase, can be used to upgrade a nodule to LR-4 (probably HCC), but not to LR-5 (definitely HCC). On the other hand, ancillary features that favor benignity can also be used to downgrade the LR category.

There is a tendency of incorporating HCA in the evaluation of cirrhotic patients^(50)^ and the latest LI-RADS version added it as an ancillary feature^(47)^. A recent study from Chen et al.^(51)^ evaluated the value of hypointensity on hepatobiliary phase imaging of gadoxetic acid in the 2014 version of the LI-RADS. They showed that the use of hepatobiliary phase imaging from gadoxetic acid as an additional criteria improved the sensitivity of LI-RADS to distinguish HCCs from benign hepatic lesions, while maintaining high specificity.

### Assessment of tumor response on MRI after locoregional therapy

With increasing comorbidities associated with patients with cirrhosis, there has been an evolution of minimally invasive approaches to treat HCCs. Percutaneous and laparoscopic radiofrequency ablation and microwave ablation are thermoablative therapies that are now widely used to treat small HCCs^(52,53)^. Successful ablation could achieve survival rates comparable to surgical resection^(54)^. Determination of ablation success is critical, as residual or recurrent tumor can potentially be re-ablated, and early retreatment is associated with better outcome.

**Figure 10:**
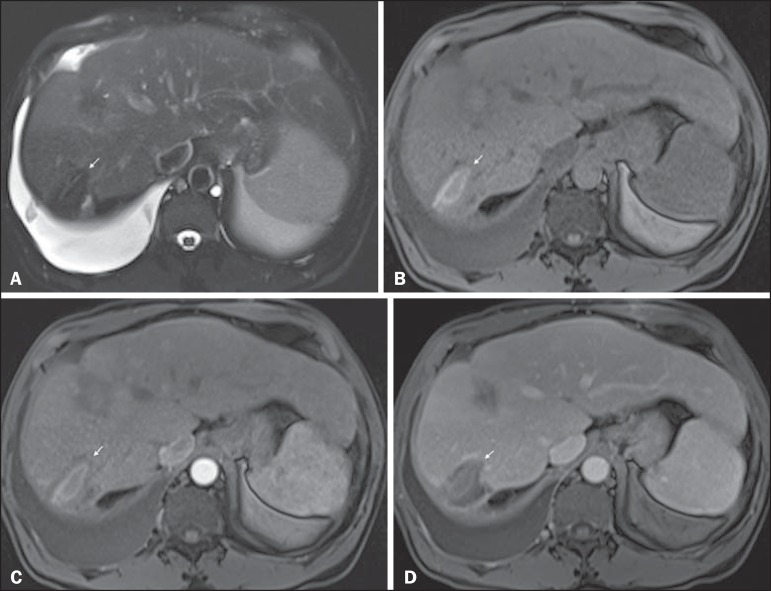
*Post-microwave ablation of HCC in a patient with chronic hepatitis C.* Axial fat-suppressed SS-FSE T2-WI (**A**), axial pre- (**B**) and postcontrast fat-suppressed 3D-GRE T1-WI in the arterial (**C**) and interstitial (**D**) phases. The treated area is seen on the right hepatic lobe (arrow, **A–D**), showing low-signal intensity on T2-WI (**A**) and a rim of high-signal intensity on pre-contrast T1-WI (**B**). On dynamic postcontrast images, the treated lesion shows no enhancement along all postcontrast dynamic phases (**C,D**), i.e., the high-signal intensity rim is identical to that shown on unenhanced image (**B**), so it represents persistent high intrinsic T1 signal rather than enhancement. This feature is consistent with absence of residual viable neoplasm.

**Figure 11:**
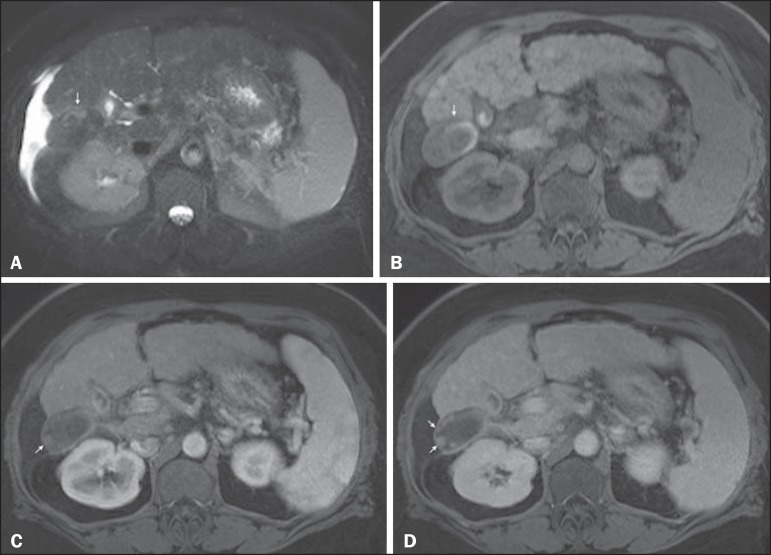
*Recurrence of HCC treated by radiofrequency ablation.* Axial fat-suppressed SS-FSE T2-WI (**A**), axial pre- (**B**) and post-contrast fat-suppressed 3D-GRE T1-WI in the arterial (**C**) and interstitial (**D**) phases. A treated area is seen at the right lobe (arrow, **A,B**), showing iso-signal intensity on T2-WI (**A**) and a partial/ interrupted rim of high-signal intensity on pre-contrast T1-WI (**B**). On the dynamic postcontrast images, progressive peripheral nodular enhancement is evident (arrows, **C,D**). These features are consistent with recurrence of disease.

**Figure 12:**
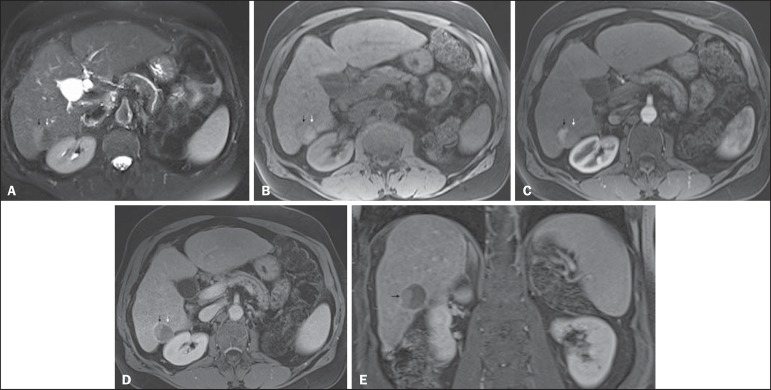
*Recurrence of HCC after chemoembolization.* Axial fat-suppressed SS-FSE T2-WI (**A**), axial pre- (**B**) and postcontrast fat-suppressed 3D-GRE T1-WI in the arterial (**C**) and interstitial (**D**) phases, and coronal postcontrast fat-suppressed 3D-GRE T1-WI in the interstitial phase (**E**). A treated area post-TACE is seen in the right liver lobe (arrows, **A–E**), showing heterogeneous intensity on T2-WI, with areas of moderate high-signal (black arrow, **A**) and low-signal (white arrow, **A**) intensity. These same areas show low-signal (black arrow, **B**) and high-signal (white arrow, **B**) intensity on precontrast T1-WI, respectively. On the dynamic postcontrast images, the areas of high-signal T2-WI are hypervascular (black arrow, **C**) and show washout and pseudocapsule on interstitial phase (black arrow, **D,E**), consistent with residual/recurrent HCC. The medial aspect showed no signs of recurrence.

In patients with advanced HCC (without vascular invasion or extrahepatic spread), transarterial chemotherapy is the only treatment that has proved to extend life expectancy^(55)^. Regardless of the treatment modality, the best indicator of successful ablation is the absence of enhancement on postcontrast images (Figure 10)^(56)^. Due to its high soft tissue contrast and high sensitivity to intravenous gadoliniumbased contrast agents, MRI plays an important role in the evaluation of therapeutic response of HCC following ablative techniques (Figure 11). The ablation cavity occasionally appears T1 hyperintense and subtracted images better show the presence or absence of enhancement in this instance. Although a thin, smooth rim of hyperemic reactive tissue persists around the ablated cavity for several months, nodular or mass-like internal or perilesional enhancement suggests residual or recurrent tumor^(56)^ (Figure 12). It should be noted that following thermoablation procedures, residual or recurrent tumor might not hyper-enhance on the first 2 months after treatment^(57)^; however, hyper-enhancement usually occurs on subsequent MRIs. Lack of hyperintensity on DWI and evidence of regression of restricted diffusion on ADC maps supports successful tumor ablation^(58)^.

## CONCLUSION

MRI is the mainstay of noninvasive evaluation of the cirrhotic liver. The sensitivity of arterial phase hyper-enhancement and delayed washout is recognizable relatively low for HCCs < 20 mm. In the second part of this review, we described new techniques and the utilization of hepatobiliary contrast agents along with the ancillary MR imaging features that appear to improve the sensitivity of HCC detection, which may potentially modify patients' management.

MRI has been shown to be superior to CT, not only in the diagnosis of HCC, but also in the assessment of tumor response following therapy. In this review, we shortly described the most important MRI aspects that radiologist should be aware of when assessing tumor response after locoregional therapy.
